# The Effect of Tuning Cold Plasma Composition on Glioblastoma Cell Viability

**DOI:** 10.1371/journal.pone.0098652

**Published:** 2014-05-30

**Authors:** Xiaoqian Cheng, Jonathan Sherman, William Murphy, Edward Ratovitski, Jerome Canady, Michael Keidar

**Affiliations:** 1 Department of Mechanical and Aerospace Engineering, The George Washington University, Washington, D.C., United States of America; 2 Department of Neurosurgery, The George Washington University, Washington, D.C., United States of America; 3 Department of Electrical and Computer Engineering, The George Washington University, Washington, D.C., United States of America; 4 Head and Neck Cancer Research Division, Johns Hopkins University School of Medicine, Baltimore, Maryland, United States of America; 5 Institute for Advanced Biological and Technical Sciences, USMI, Takoma Park, Maryland, United States of America; University Paul Sabatier, France

## Abstract

Previous research in cold atmospheric plasma (CAP) and cancer cell interaction has repeatedly proven that the cold plasma induced cell death. It is postulated that the reactive oxygen species (ROS) and reactive nitrogen species (RNS) play a major role in the CAP cancer therapy. In this paper, we seek to determine a mechanism of CAP therapy on glioblastoma cells (U87) through an understanding of the composition of the plasma, including treatment time, voltage, flow-rate and plasma-gas composition. In order to determine the threshold of plasma treatment on U87, normal human astrocytes (E6/E7) were used as the comparison cell line. Our data showed that the 30 sec plasma treatment caused 3-fold cell death in the U87 cells compared to the E6/E7 cells. All the other compositions of cold plasma were performed based on this result: plasma treatment time was maintained at 30 s per well while other plasma characteristics such as voltage, flow rate of source gas, and composition of source gas were changed one at a time to vary the intensity of the reactive species composition in the plasma jet, which may finally have various effect on cells reflected by cell viability. We defined a term “plasma dosage” to summarize the relationship of all the characteristics and cell viability.

## Introduction

Recent development in physics research has lead to the production of cold atmospheric pressure plasma, a type of plasma that is formed at relatively “cold” temperatures, or room temperature. Previously, plasma was limited in application by its properties, including high voltage and high temperatures, but this most recent innovation has moved the focus to possible biological applications including food disinfection, wound healing, surgical procedures and even cancer treatment [1] [2]. The treatment of living tissues by cold plasma can be classified into two approaches: dielectric barrier discharge (DBD) and plasma jet. DBD is generated at a high voltage between two electrodes in the air, with at least one electrode being insulated in order to prevent current build-up, creating electrically safe plasma without substantial gas heating [3]. In the case of DBD, all the generated agents have direct contact with the treated sample. Plasma jets, on the other hand, also have high voltage between two electrodes, generate plasma inside of a quartz tube [4] and treat biological samples remotely. In this study, a helium plasma jet is employed because it produces a stable, homogenous and uniform discharge at atmospheric pressure, and it operates without a dielectric cover over the electrode, yet is free from filaments, streamers and arcing [5].

Cancer is a vast collection of diseases that share a common devastating similarity of unrestricted cell growth, however similarity in cell functions and metabolism between normal and tumor tissues creates serious obstacles in the specific ablation of tumor tissue, while leaving normal tissue intact and unharmed. To improve efficiency and safety of anti-cancer therapies the researchers and clinicians alike are prompted to develop targeted combined therapies that especially minimize damage to healthy tissues while eradicating the body of cancerous tissues.

In the recent decade, astounding phenomenon between both cold plasma types and biological tissues has spurred a new era of innovation in an emerging field called plasma medicine [2]. Various bacterial, such as Geobacillus stearothermophilus, bacillus cereus, Escherichia coli [6] [7], and mammalian cell types such as neuroblastoma [8], pancreatic carcinoma [9] [10], skin carcinoma [11], lymphoblastic leukemia [12], hepatocellular carcinoma [13] [14] [15], melanoma [16] [17] [18] [19], cervical carcinoma [20] [21], lung carcinoma [22] [23] [24], bladder carcinoma [22], colon carcinoma [17], breast cancer [25], have been utilized for both in vitro and in vivo plasma therapy studies. Glioblastoma multiforme (GBM) is the most common and aggressive malignant primary brain tumor in humans, involving glial cells and accounting for 52% of all functional tissue brain tumor cases and 20% of all intracranial tumors. GBM is rare, with incidence of 2–3 cases per 100,000 individuals in Europe and North America [26]. The prognosis of this disease is generally no more than one year and the long-term survival is small [26]. Attributed to its multiformity and aggressiveness, this cancer is highly resistant to treatments including chemotherapy, radiation therapy and surgery [27]. Being the most common brain tumor [28], it garners much interest by researchers to develop novel treatments and is used in this paper as a model cell-line for treatment.

Previous research in cold atmospheric plasma (CAP) and cancer cell interaction has repeatedly proven that there is a strong connection between CAP therapy and cell death [8] [12] [22] [29]. Reactive oxygen species and reactive nitrogen species (ROS/RNS) were postulated to play a major mechanistic role in the CAP cancer therapy [8]. Intracellular ROS/RNS have been shown at low levels to induce a proliferate cell growth, whereas at high levels, above a certain threshold, cause an apoptotic arrest in cancer cells [30]. Plasma generated species (to distinguish them from the intracellular ROS/RNS, they were described as extracellular ROS/RNS) may be delivered into the cells directly by the media, stimulating the cells to produce a massive amount of intracellular ROS/RNS, or they react with the culture media to form other reactive species. Either one or both of the two cases generate great oxidative stress to cells, leading to cell death. The mechanism for this exact interaction, however, is not fully understood and not conclusively proven. The basic way to understand the mechanism by which CAP exerts its effect on tumor cells is to figure out the plasma jet composition. In this study, a thorough understanding of how each species of the CAP treatment impacts cell viability is provided. CM_H_2_DCFDA (Life Technologies), an indicator for reactive oxygen species in the cells, was utilized to demonstrate how intracellular ROS level varies with the different plasma composition (extracellular ROS/RNS level). In order to determine the threshold of plasma treatment on U87, normal human astrocytes (E6/E7) were used as the comparison cell line. The CAP therapy is multifaceted in the way it produces “plasma dosage” including treatment time, voltage, flow-rate and plasma-gas composition. This study is designed to determine a chemical mechanism of CAP therapy through an understanding of the composition of the plasma and conditions of its usage.

## Materials and Methods

### 1. Cold Atmospheric Plasma Configuration

**Figure 1:**
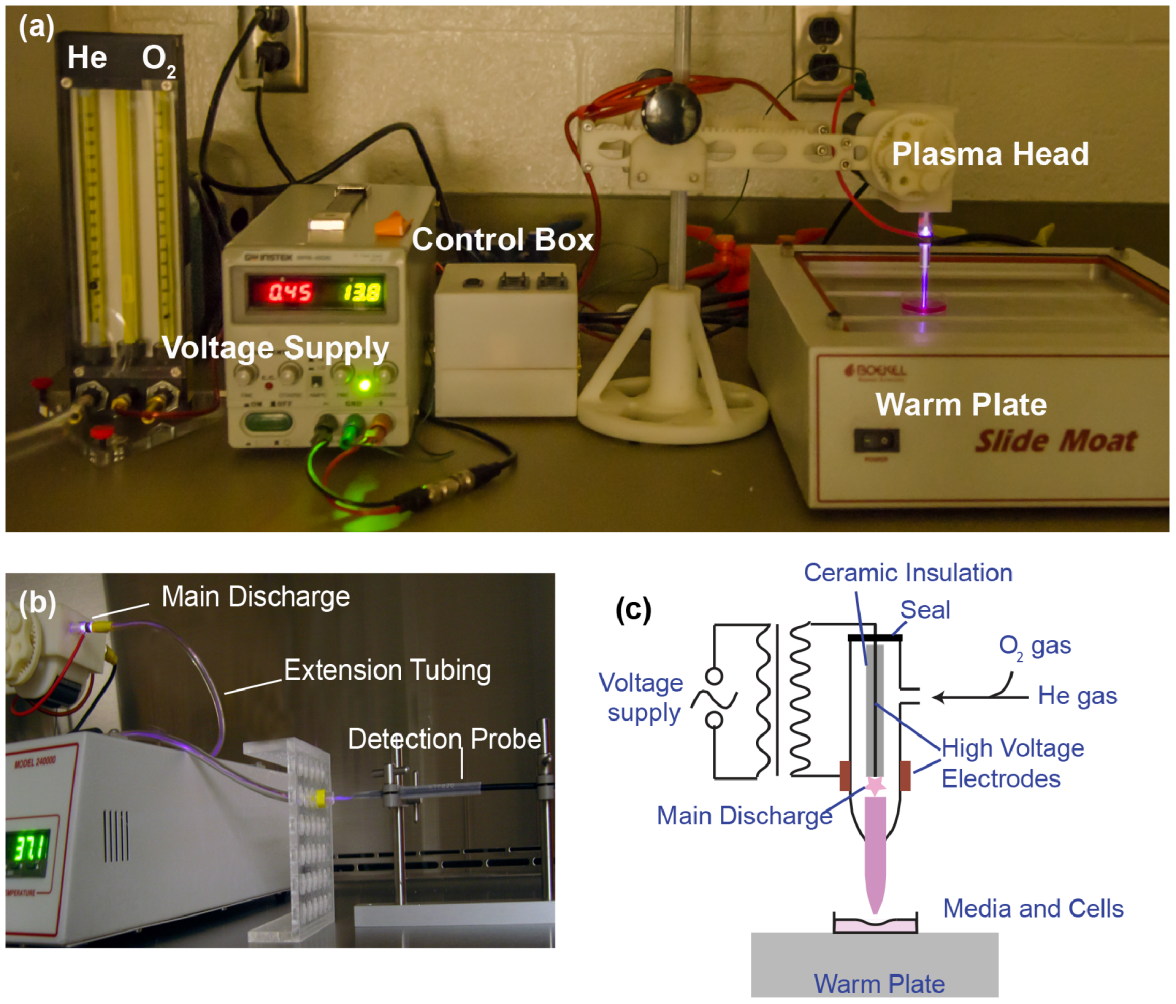
(a) The cold plasma device setup: voltage supply, control box, plasma head, and flow meter. (b) The extension tube was attached on the nozzle to eliminate the sparks near the central electrode. Optical probe was placed in front of the extension at a distance of ∼2.5 cm, same as the real treatment distance between the plasma nozzle and the cells (c) Schematic image of the CAP device.

The cold plasma device created at the George Washington University has a configuration of central powered electrode of 1 mm diameter and a grounded outer electrode wrapped around the outside of a 4.5 mm diameter quartz tube (Figure 1). The electrodes were connected to a secondary of high voltage resonant transformer (voltage up to 10 kV, frequency ∼30 kHz) [4]. The plasma discharge was driven by AC high voltage. Electrical measurements were performed with a Tektronix TDS3014C Digital Phosphor Oscilloscope. Emission spectra were recorded with an optical fiber which was connected to a fiber optic spectrometer (EPP2000-HR, Stella Net, measurements can be made in UV-VIS-NIR ranges from 190–2200 nm). The feeding gas was helium and helium/oxygen mixture. In order to show the combination impact of flow rate, gas content, output voltage, incubation time after treatment, and treatment duration, cells were placed into three 96-well plates. The helium flow rate was within the range of 2∼6 l/min; when the helium (He)/oxygen (O_2_) mixture as the feeding gas, the oxygen percentage was 0 ∼ 0.63% (v/v). The output voltage was set to 3.16 kV.

### 2. Optical Emission Spectroscopy

In the present work, a range of wavelength 250–850 nm, i.e. UV-visible-NIR, was investigated on CAP jet to detect various ROS and RNS (atomic oxygen [O], hydroxyl radical [-OH], nitric oxide [-NO], nitrogen [N_2_], and nitrogen cation [N_2_^+^]). The spectrometer and the detection probe were purchased from Stellar Net Inc. Instead of pointing the probe directly to the nozzle of the CAP jet, an extension tube was used to prolong the jet to eliminate the emission of the discharge near the central electrode (Figure 1). The discharge area produced was of great concern in spectroscopic studies of the plasma jet since it was located at a significant distance from the treatment area. As such, a setup was devised to minimize the measurement of the discharge area and solely focus on the afterglow. Under normal treatment conditions, used in this research, the afterglow was the area of most importance to study.

Following this logic, an extension tube was created that had a central electrode to guide the cold atmospheric pressure plasma jet’s afterglow a distance away from the plasma jet to be studied. This was far enough away from the discharge so as not to factor in the excess electromagnetic radiation produced. Per experimental design, the fiber optical spectrometric device was parallel to the extended jet and in directly in line with the afterglow. This is the most logic measurement approach, since the cells are treated directly under the jet. The extension tube was sealed tightly around the discharge area of the tube. A secondary central electrode was used to propagate the plasma afterglow a sustainable distance away from the discharge area. The plasma afterglow was measured in line-of-sight of the jet by the fiber optical spectrophotometric device. The optical probe was place at a distance of ∼2.5 cm in front of the extension tube, coherent with the real distance between the plasma jet nozzle and the cells during treatment. This is a critical precision because the spectrum of the jet will vary with the specific measure point, and the content of ambient air such as N_2_, O_2_ and H_2_O will affect the intensity of oxygen, nitrogen, and hydroxyl radicals [31] [32]. Integration time of the collecting data was set to 100 ms.

### 3. Cell Culture

The human brain glioblastoma cancer cell line, U87, and the normal human astrocytes, E6/E7, were used in this study. Both cell lines were cultured in Dulbecco’s Modified Eagle Medium (Life Technologies) supplemented with 10% (v/v) fetal bovine serum (Atlantic Biologicals) and 1% (v/v) Penicillin and Streptomycin (Life Technologies). Cultures were maintained at 37°C in a humidified incubator containing 5% (v/v) CO_2_. Cells were observed under a Nikon Eclipse TS100 inverted microscope.

### 4. Cell Viability Assay

Cell viability was monitored using the MTT assay (Sigma-Aldrich, M2128), which is a colorimetric assay for measuring the activity of mitochondria and cellular dehydrogenase enzymes that reduce 3-[4, 5-dimethylthiazol-2-yl]-2, 5-dyphenyltetrazolium bromide, MTT, to its insoluble formazan, giving a purple color. Cells were plated into three 96-well flat-bottomed microplates (Falcon) in 100 ul medium per well. Confluence of each well was ensured to be at ∼15–20%. Cells were then incubated for one day to ensure a proper cell adherence and stability. Cells were rinsed with phosphate buffered saline (PBS) replaced with a fresh medium, and treated with CAP followed by an additional incubation at 37 C for 24, 48, and 72 h. Cells were then rinsed with PBS, and 100 ul of MTT solution per well (7 mg Thiazolyl Blue Tetrazolium Blue in 10 ml medium for one plate) was added into each well. Reactions were maintained for 3 h at 37°C. The MTT solution was aspirated and 100 ul of MTT solvent (0.4% (v/v) HCl in anhydrous isopropanol) was added to each well to dissolve formazan crystals. Reactions were monitored by the Synergy H1 Hybrid Multi-Mode Microplate Reader at 570 nm. Each one of the conditions (voltage, flow rate, oxygen fraction, treatment time, and incubation time after treatment) was applied to 12 wells in one trial. The entire set of experiments was repeated three times in triplicate. This made the sample size 36.

### 5. Intracellular ROS (General) Measurement and Peroxynitrite Measurement

5, 6-Chloromethyl-2′, 7′-dichlorodihydrofluorescein diacetate (CM-H_2_DCFDA) was purchased from Invitrogen for the general intracellular ROS measurement. U87 cells were plated in 35 mm glass bottom culture dishes (MatTek Corporation). Detailed protocol can be found on the Invitrogen website. Briefly, 24 h after the plating, the medium was aspirated, and CM-H_2_DCFDA solution (8.5 nM) was added. Afterwards, the cells were treated with helium cold plasma under various conditions. 30 min later, fluorescence images were obtained by the Laser Scanning Confocal microscopy (LSCM, Zeiss, LSM 510) at 40x magnification. For images with CM-H_2_DCFDA, an excitation wavelength of 488 nm and emission wavelength of 515 nm were used. Quantification of the fluorescence intensities was performed by the official software Zen 2012 lite.

Peroxynitrite (ONOO-) detection kit was purchased from Cell Technology. The experiment was performed exactly according to the detailed protocols given on the official website. Cells were plated in black clear-bottom 96 well plates (purchased from Costar). The final solution of the dye added into the cells was 5 nM. After loaded with the dye, the cells were treated with plasma under various helium flow rate. The fluorescence intensity of the ONOO- was obtained with a microplate reader (Synergy H1 Hybrid Multi-Mode) 1 h after the plasma treatment.

### 6. Statistical Analysis

Results were plotted using a Microsoft Excel software (2010 for Windows) as mean ± standard deviation. Student t-test was used to check the statistical significance (*p<0.05, **p<0.01, ***p<0.001).

## Results and Discussion

**Figure 2:**
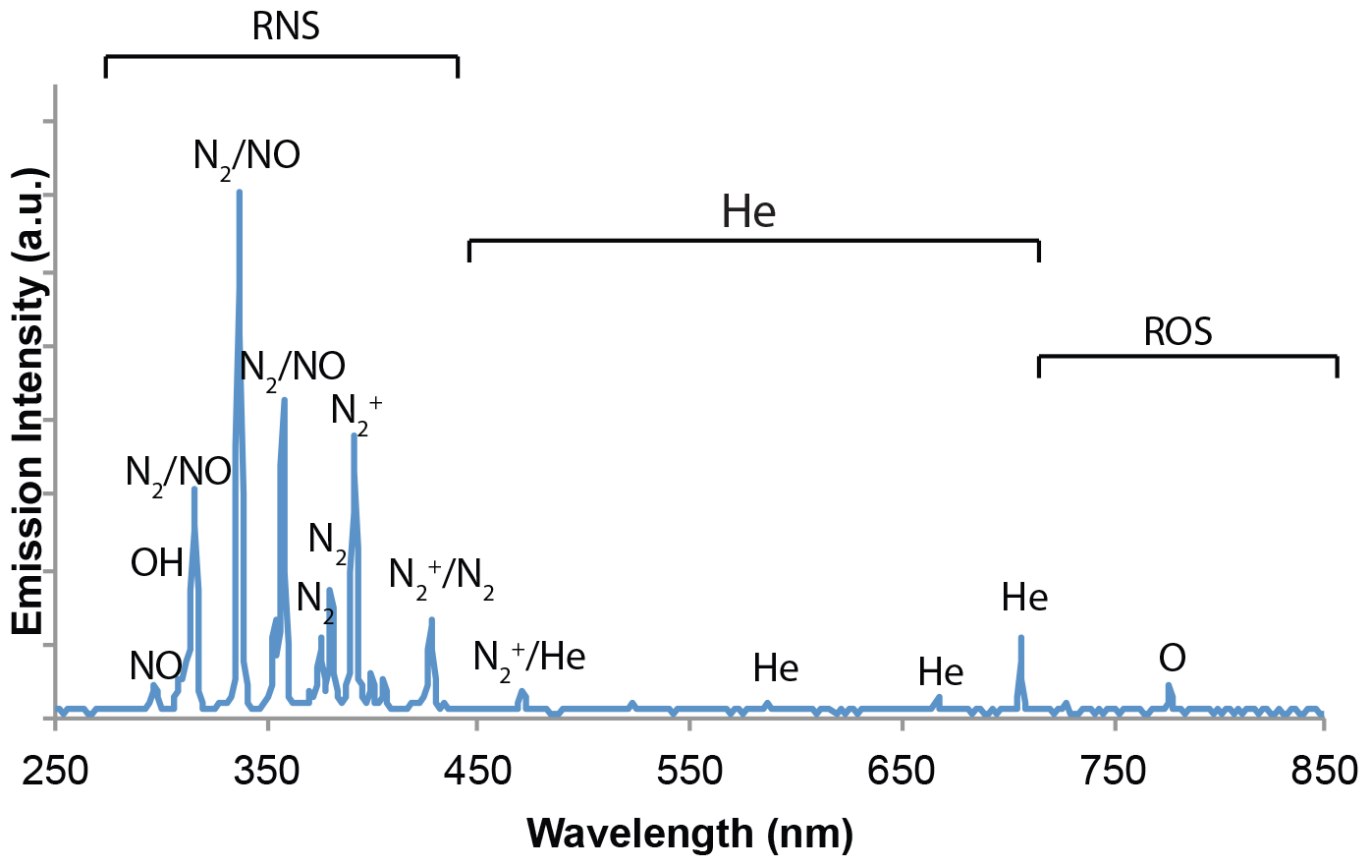
Typical spectrum of helium plasma jet (measured at output of 3.16 kV and helium flow rate at 4.7 l/min). The determination of major reactive species is shown.

Figure 2 shows a typical spectrum of helium cold plasma jet interacting with the ambient air with output voltage of 3.16 kV and a helium flow rate of 4.7 l/min. The identification of emission lines and bands was mainly according to [33]. In the special range of 250–300 nm, there are very weak emission lines, which are detected as NO lines [34]. Their magnitudes are at most a few thousandths of the highest peak N_2_^+^ (391 nm). Repeated measurements (data not shown) were performed to confirm the result. As previously shown, the inactivation effect on bacteria by UV-radiation is mostly related to the DNA/RNA damage in UV wavelength of 200–280 nm [35]. Thus, it can be concluded that UV photons are not major CAP species with the experimental setup. The features were assigned as helium (He) lines between 550–750 nm.

Atomic oxygen (O) (including the ground state and all the excited states of atomic oxygen) is believed to have a significant effect on cells and therefore a broad biomedical application. The species lines between 300–500 nm are still not clearly determined [34] [36]. Species at the wavelengths of 316, 337, 358 nm could be defined as N_2_^3^Π or NO β ^2^Π (denoted as N_2_/NO in the following text) according to [33], because both of the two species have possible optical emission at these wavelengths. The cold plasma jet is a complicated environment that combines the comprehensive effect of different ions and neutrals. It is shown that O (777 nm), OH (309 nm), N_2_^+^ (391 nm), N_2_ or NO lines (316, 337, 358, 427.5 nm) are the domain species of the spectra.

### 1. Voltage Variation Effect

**Figure 3:**
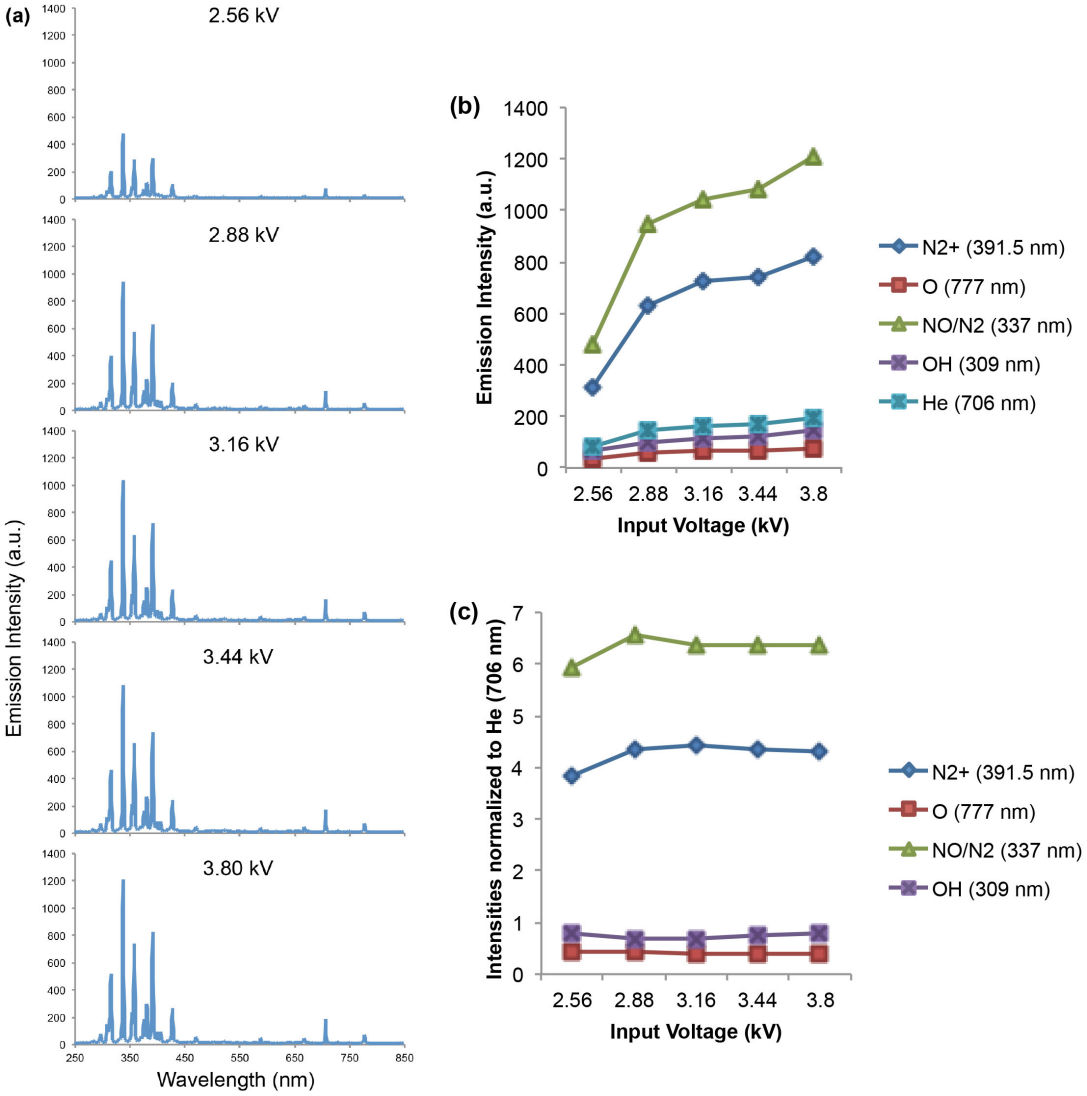
(a) The increase in output voltage from 2.56 to 3.8 kV led to the corresponding rise in the intensity of each species. (b) A closer look at the increasing trend of major ROS and RNS species with voltage increasing. (c) The increasing of each species is proportionally to the output voltage increasing from 2.56 to 3.8 kV when normalized to He (706 nm).

The increase in output voltage from 2.56 to 3.8 kV led to the corresponding rise in the intensity of each species (Figure 3a). Figure 3b gives a closer look at the increasing trend of major ROS and RNS species. Helium line at 706 nm is known to represent energetic electrons [37], and recently it has been linked to energetic electrons in RF atmospheric plasmas [38] [39]. Hence, the intensities of the major species were normalized to He (706 nm), shown in Figure 3c. The flat lines demonstrate that the increasing of each species is proportionally to the output voltage increasing from 2.56 to 3.8 kV.

### 2. Plasma Treatment Duration Effect

#### A. Cell viability

**Figure 4:**
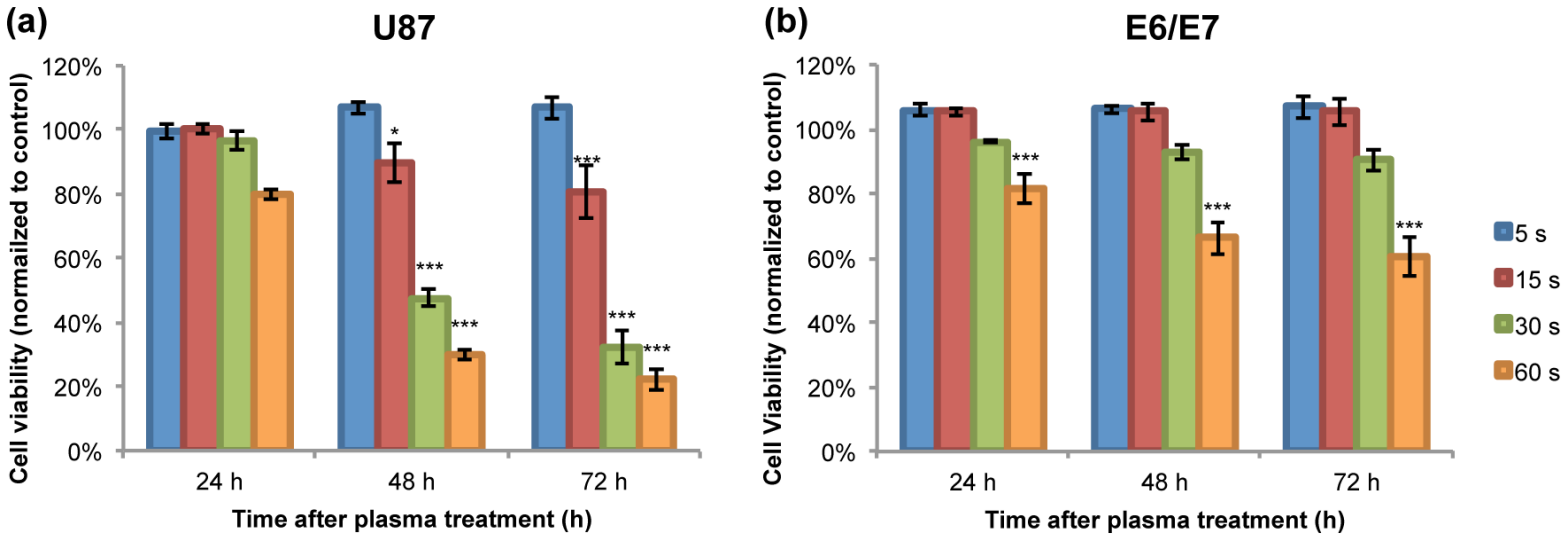
(a) 24, 48, 72 h MTT assay results of U87 treated with 5–60 s duration of helium plasma jet. (b) 24, 48, 72 h MTT assay results of E6/E7 treated with 5–60 s duration of helium plasma jet.

To find out the duration time needed for helium plasma therapy of U87 cells, the normal human astrocytes E6/E7 were used as contrast cell line. Both cancer and normal cells were treated for various durations from 5 sec to 60 sec. The results of the MTT assay (Figure 4) showed that after 72 h, around 80% of U87 cells died at 30 sec and 60 sec of plasma treatment, while E6/E7 cells remained 90% and 60% cell viability at 30 sec and 60 sec plasma treatment at 72 h post-treatment time point. The p values for both cell lines after 60 sec treatment after 72 h showed statistically significant compared to control, while the p value for 30 sec plasma treated E6/E7 was not (Figure 4). So the threshold needed for U87 cells is determined as 30 sec. Normally a LD_50_ (median lethal dose) data should be presented to determine a threshold of a drug or a physical agent, but in this special case, normal cells remained their viability above 50%, while viability of 30s- and 60s- plasma treated U87 cells dropped below 50% less than 48 h (Figure 4). All MTT assays of the other characterization of plasma composition (source gas flow rate, source gas composition) were based on this threshold: plasma treatment duration was kept at 30 sec per well.

### 3. Helium Flow Rate Variation Effect

#### A. On spectrum

**Figure 5:**
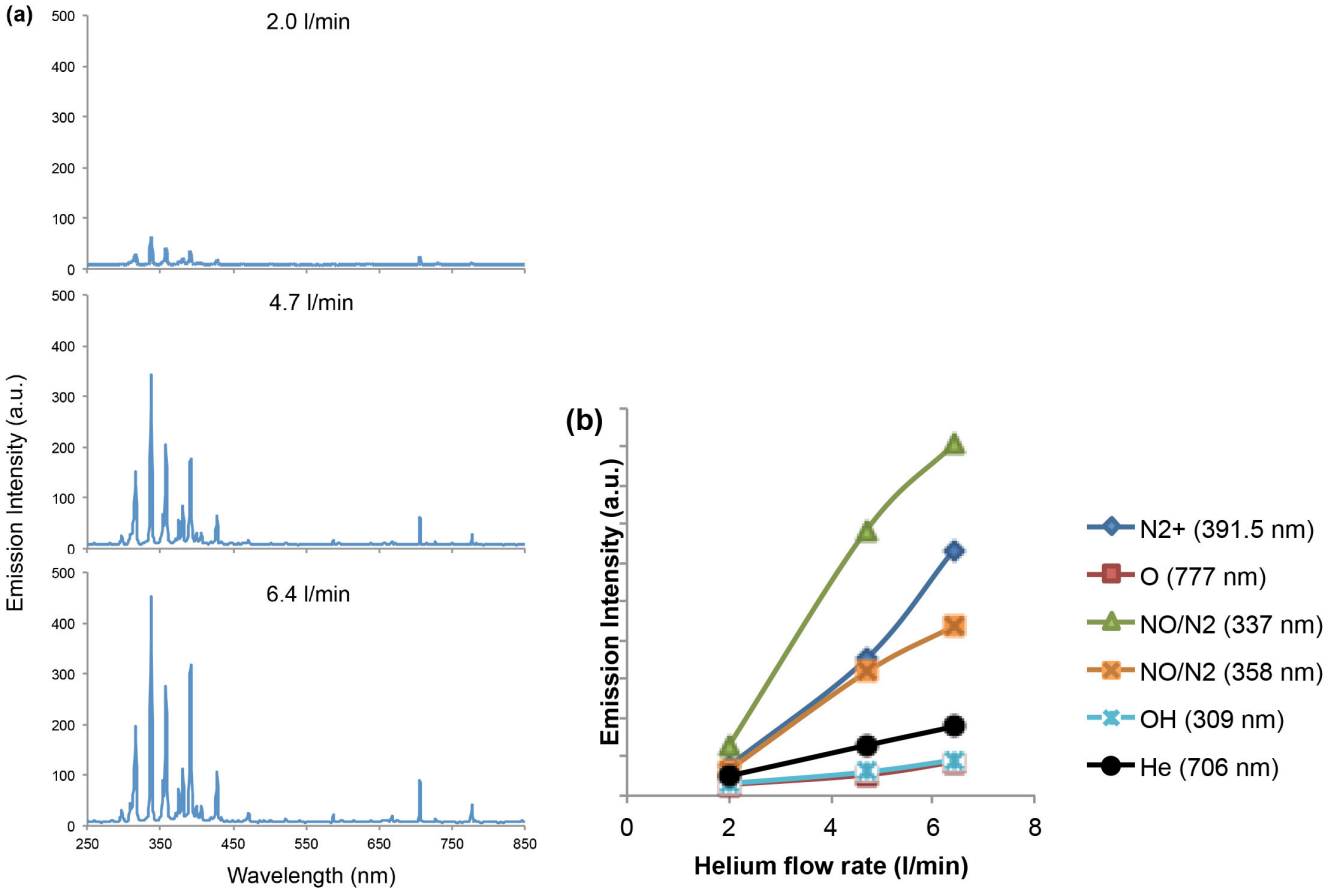
(a) The spectrum of helium plasma jet with flow rate of 2.0, 4.7 and 6.34 l/min. (b) The trend of major RNS and ROS with helium flow rate change at 2.0, 4.7, and 6.4 l/min.

Three values of helium flow rate were chosen to treat the U87 cells: 3.15, 4.70, 6.34 l/min, while the output voltage was kept at 3.16 kV. The trend of major ROS and RNS increasing with helium flow rate were depicted in Figure 5a. We showed that the main increase occurred at the wavelength of 300∼500 nm, namely, RNS (Figure 2). Figure 5b more intuitively demonstrated that the NO/N_2_ (337 nm, green), NO/N_2_ (358 nm, purple), and N_2_^+^ (391 nm, blue) increased faster than OH (309 nm, orange) and O (777 nm, red).

#### B. On cell viability

**Figure 6:**
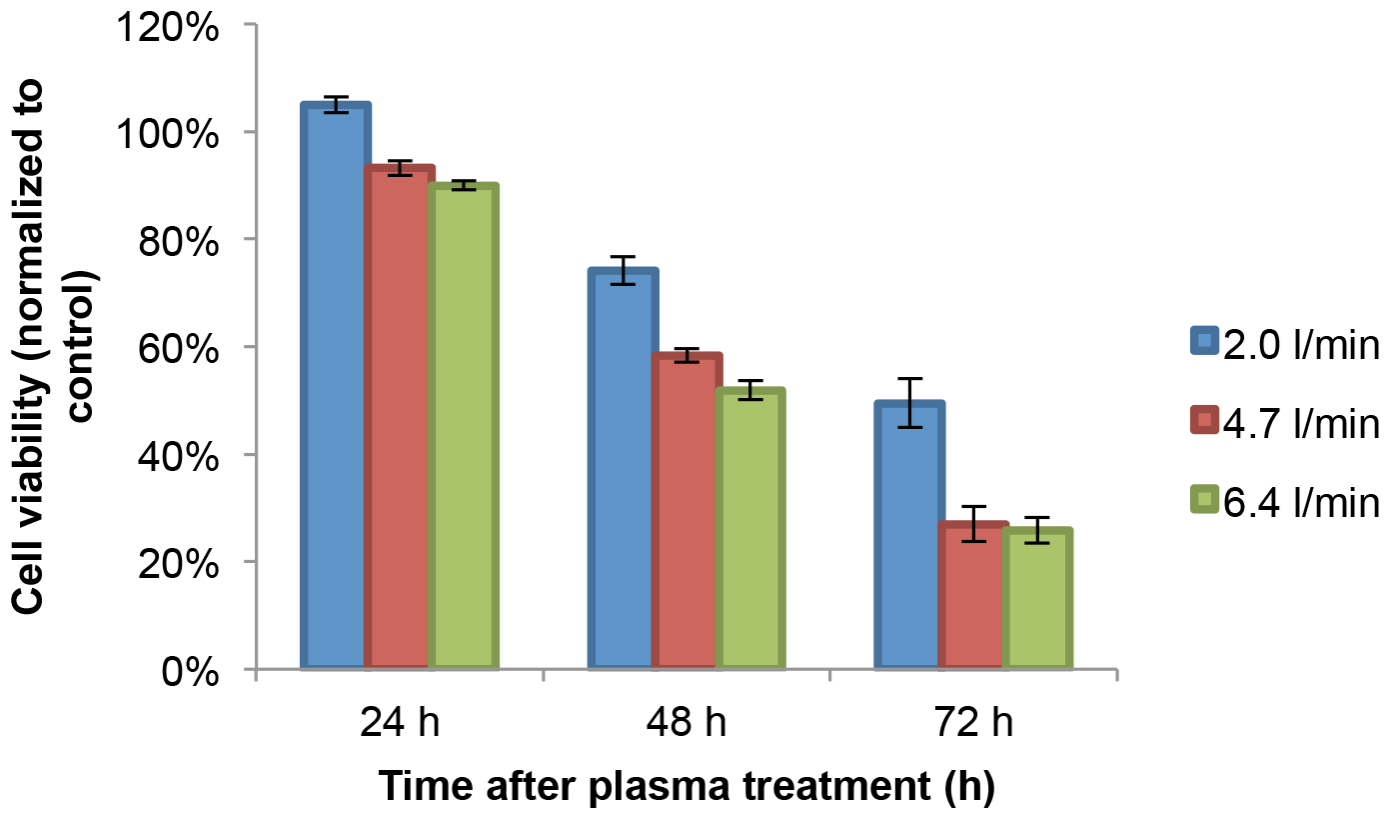
24, 48, 72–6.34 l/min helium flow rate.

The same setup and conditions of various helium flow rate plasma as used in spectrum measurement were used on U87 cells cultured in 96 well plates, as described in Materials and Methods section. Each well was treated for 30 s. The results from the MTT assay showing the effect of helium flow rate on the cell viability were depicted in Figure 6. The MTT assays were performed at 24, 48, 72 h after treatment under different conditions. All cell viability data were normalized relative to control. Figure 5 and 6 demonstrated that the higher the helium flow rate is, the higher ratio of RNS/ROS was observed, and the cell viability decreased. This suggests that RNS generated exogenously by the CAP device more significantly contribute to the survival of U87 cancer cells under the CAP treatment than exogenous ROS.

**Figure 7:**
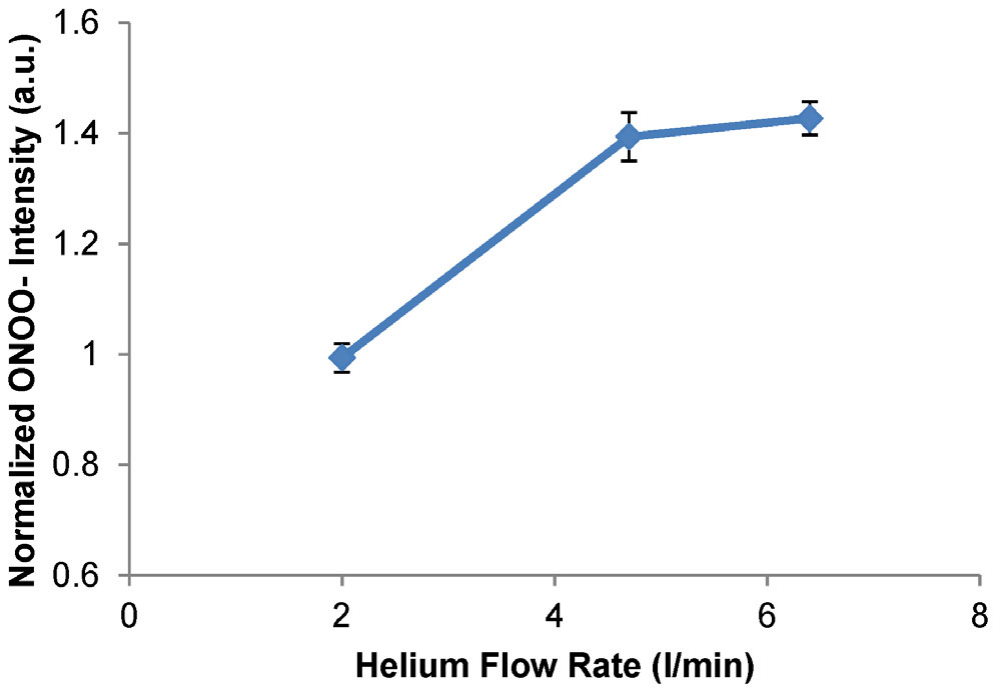
Intracellular peroxynitrite intensity measured in U87 after plasma treatment with various helium flow rate. Measurement was performed at 1-plasma treatment.

It is well-known that NO is an omnipresent intercellular messenger in all vertebrates, modulating blood flow, thrombosis, neuronal activity, immune response, inflammation, and plays a critical role in tumorigenesis by modulating the apoptotic machinery [13] [14] [15] [16]. According to Pacher and co-workers, NO and superoxide (O_2_^−^) can easily form peroxynitrite (ONOO^−^) once they collide or even locate within a few cell diameters of each other [40]. Peroxynitrite is a powerful oxidant and nitrating agent that is known to be a much more damaging to the cells than NO or superoxide, because cells readily remove superoxide and NO to reduce their harmful effects, while fail to neutralize peroxynitrite [41]. A major limitation to the acceptance of peroxynitrite or any RNS as a significant player in disease was whether enough could be produced to exert a sufficient damage to tumor cells. To test this notion, we used spectral imaging showing that CAP with helium gas produced both RNS and ROS exogenously, with the former appear in higher concentrations (Figure 5). This is the perfect condition for peroxynitrite formation when directly applied to a cellular environment. As shown in Figure 7, the intensity of ONOO- increases with helium flow rate. The measurement of ONOO- was performed at 1 h time point after the helium plasma treatment with various flow rate. The rate for peroxynitrite synthesis increases drastically with a modest increase of the simultaneous production of superoxide and NO. According to Lukes et al, the formation of NO_2_·, NO· and OH· radicals and NO+ ions by the discharge of plasma are at the gas-liquid interface and in the liquid [42]. Consequently, the generation of a moderate flux of peroxynitrite over long periods of time would result in substantial oxidation and potential destruction of host cellular components leading to a deregulation of critical cellular processes, disruption of cell signaling pathways, and induction of the cell death through both apoptosis and necrosis [43]. This can be an explanation why the cell viability did not drop much during the first 24 h after the CAP treatment, however decreased dramatically after 72 h (Figure 6).

### 4. Oxygen Fraction Effect

#### A. On spectrum

**Figure 8:**
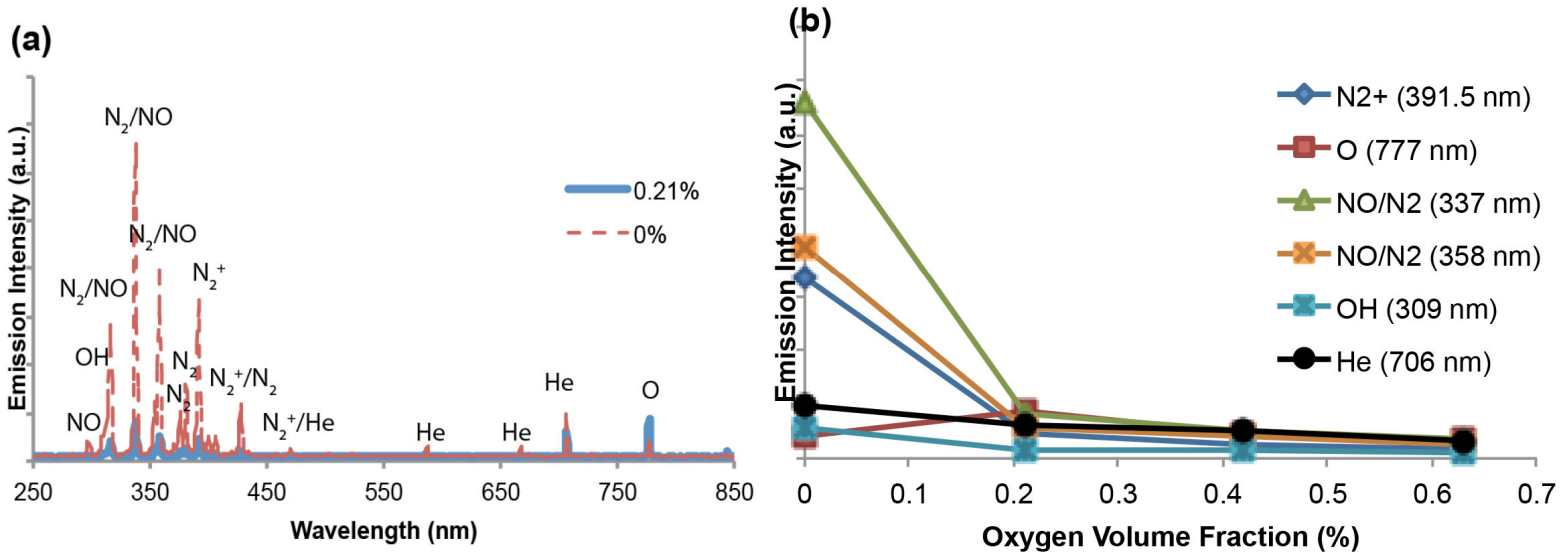
(a) Comparison between spectrum of He/O_2_ mixture plasma jet and He plasma jet at output voltage 3.16 kV. (b) The trend of major plasma generated RNS and ROS with oxygen volume fraction change at 0, 0.21, 0.42, 0.63%.

Figure 8 shows the volume fraction of oxygen’s impact on the spectrum of the mixture plasma jet. The experimental setup was chosen as 0.21%, 0.42%, and 0.63% for oxygen fraction in the mixture gas, while the other conditions were kept the same (He flow rate remained at 4.7 l/min; output voltage remained at 3.16 kV). The addition of oxygen into the plasma gas supply makes the ionization extremely difficult. The plasma plume dampened very quickly even with a tiny bit of oxygen fraction increase. As shown in Figures 8a and 8b, the addition of a small amount of oxygen (from 0% to 0.21%) resulted in a rapid damp of the jet, especially the species in the range of 300∼450 nm, while RNS almost disappeared in mixture gas plasma (blue) compared to helium gas plasma (red). Although the intensity of every species decreased significantly with the addition of oxygen, the latter was the only species that increased (Figure 8b). With more oxygen added (from 0.21% to 0.42% and 0.63%), every species decreased at the same rate.

#### B. Cell viability

**Figure 9:**
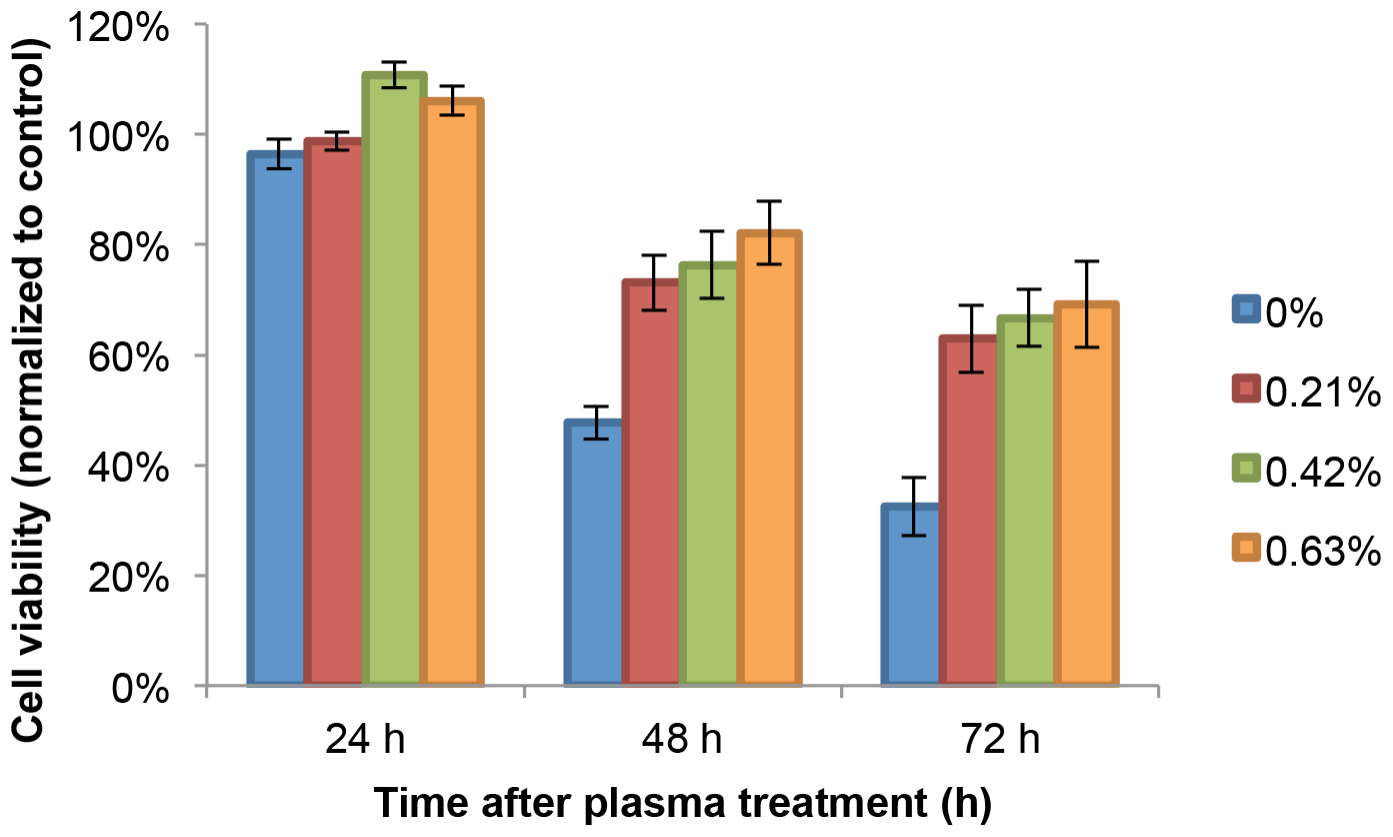
24, 48, 72–0.63% oxygen fractions in the He/O_2_ mixture gas.

We next tested the effect of oxygen volume fraction on the cell viability. The conditions used for cell treatment were exactly the same as described above in He/O_2_ mixture gas spectrum. The MTT assays were performed at 24, 48, 72 h after treatment. All viability data were normalized relative to control. Using MTT assay, we showed that the addition of oxygen into the gas supply weakened the therapeutic effect of cold plasma on the cancer cells (Figure 9). As shown in Figure 9, the cell viability increased over 60% after 72 h compared to 30% when cells were treated with the helium gas plasma jet. Along with the data presented in Figure 8 showing that every species was dampened by oxygen addition except O at 777 nm, the results of cell viability assays (Figure 9) suggest that the admixture of even the slightest amount of oxygen decreases the intensity of the cold atmospheric plasma jet, which decreased the produciton of peroxynitrite as discussed above and therefore decrease the cell death rate.

### 5. Intracellular ROS Measurement

**Figure 10:**
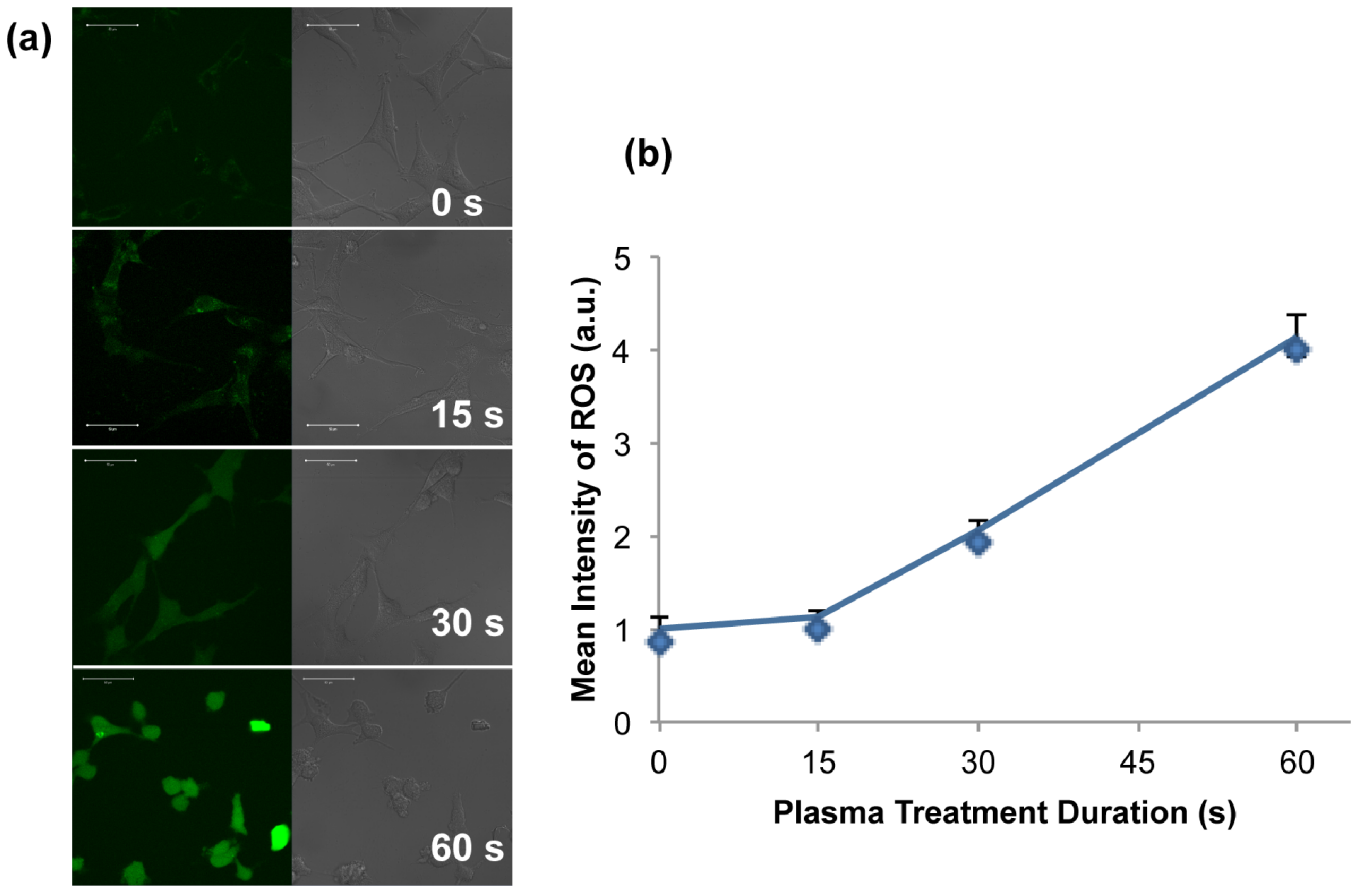
(a) Intracellular ROS generation in U87 cells under 0–60 s plasma treatment durations. Images were taken 30 min after the plasma treatment. (b) Quantification of the ROS intensity with Zen 2012 Lite.

Using the CM_H_2_DCFDA redox probe and confocal imaging, we next measured the intracellular ROS generation in U87 cells treated for various durations. The probe is non-fluorescent when chemically reduced, but after cellular oxidation and removal of acetate groups by cellular esterases the redox probe becomes fluorescent. As shown in Figure 10a, the left image section presents the fluorescent production within the cells, while the right section is the natural white channel (scale bar 50 µm). Figure 10b demonstrates the mean intensity of fluorescence as a function of plasma treatment duration. These data indicate that the longer plasma treatment was, the more amount of ROS the cells produced. But the mechanism of the ROS production, whether ROS is generated by the cold plasma jet and then directly penetrated into the cells, or the cold plasma stimulated the cells to induce the corresponding enzymes and produce endogenous ROS at a higher ratio, is not yet understood.

### CAP Dose

In summary, we varied the characteristics of the cold plasma (treatment duration, input/output voltage, flow rate and composition of feed gas) in order to obtain different major species. From the above results, we use “plasma dosage” to summarize all the variables, i.e.
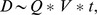
where *D* is the entire “plasma dosage” applied to the cells; *Q* is the flow rate of the feeding gas (helium); *V* is the output voltage; *t* is the treatment time.

**Figure 11:**
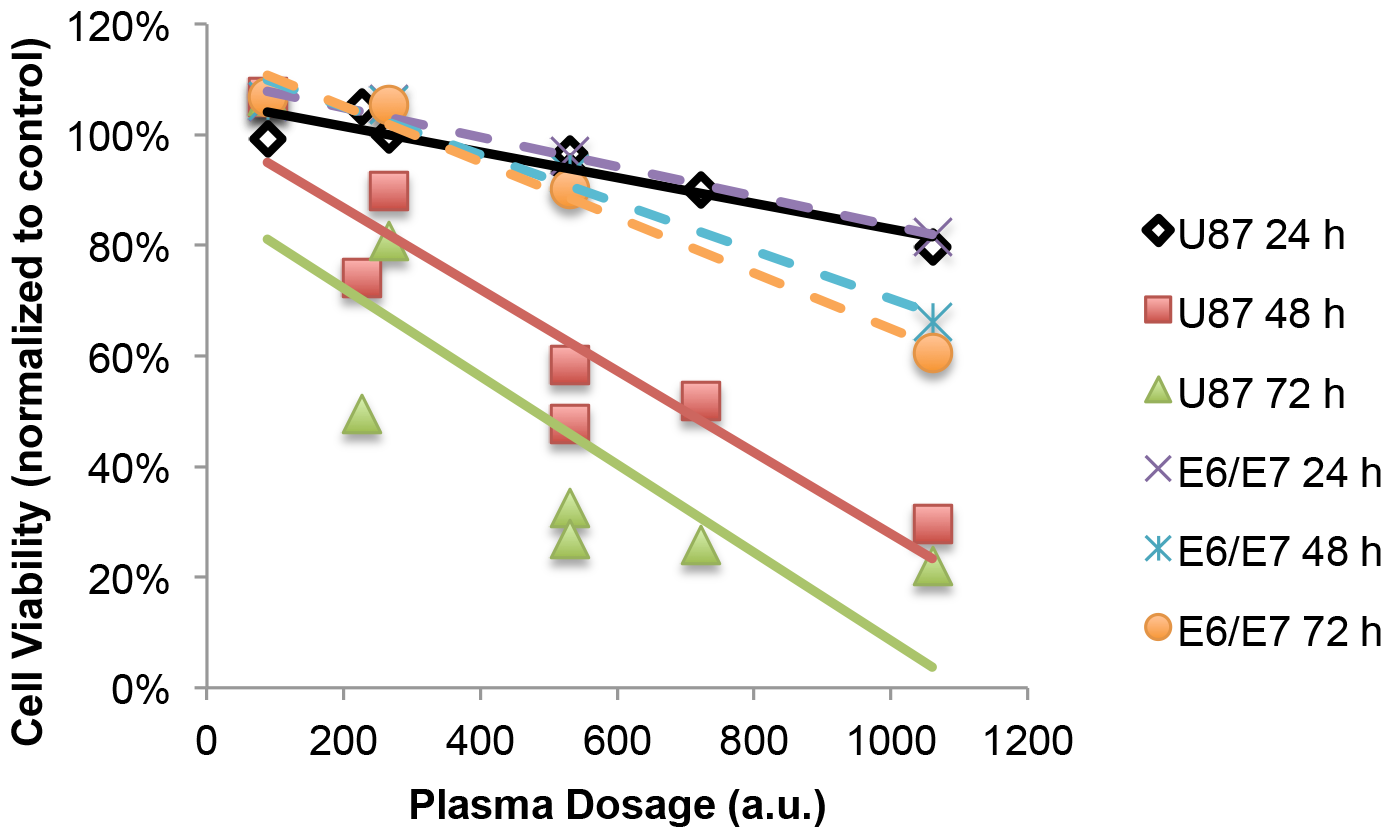
Dependence of cell viability dependence on “plasma dosage” (dash line: E6/E7 cell viability trendline of 24, 48, 72 h incubation after plasma treatment; solid line: U87 viability trendline of 24, 48, 72 h incubation after plasma treatment).

These various conditions were applied on glioblastoma U87 cells to achieve the corresponding/desirable cell death rate. To relate the cell viability to the variables, we could conclude that the higher “plasma dosage”, the more the cell viability decreased, as showed in Figure 11.

The above formula for “plasma dosage” summarized from the above experiments is exclusively applicable for our helium plasma jet. When it comes to oxygen and helium mixture gas, the ionization gets more complicated, and the intensity of the plasma jet is significantly reduced. Because of that complexity, it is difficult to determine whether atomic oxygen generated by the mixture plasma gas protects the cells or the RNS are dampened so that less peroxinitrite was produced to create oxidative stress on the cells. More experiments on the effects of specific ROS (O_2_^−^, O_3_) and RNS (NO, N_2_^+^) needed to be performed to more clearly understand the production of each species by CAP and confirm the hypothesis underlying the peroxynitrite effect on cell viability.

### Summary

It should be acknowledged that the major factors in plasma dosage are the gas flow rate, output voltage difference at the electrodes and the duration of exposure to the plasma. These three components cause a respective decrease in cell viability for cancer cells. The mechanism for which cold atmospheric pressure plasma kills cancer cells, through measurements of reactive oxygen species, can be conclusively stated to be in part by these factors. The relationship between these factors and species production is understood by the spectral measurements conducted. Each part plays a role in creating larger concentrations of reactive species: the voltage by increasing the rate of ionization, the duration by increasing the number of species that come in contact with the cancer cells and flow rate which creates a gaseous volume that facilitates better ionization. Data has already shown that increasing reactive species endogenously past a certain threshold results in apoptosis and necrosis, so increasing the “plasma dosage” results in higher exogenous reactive species which in turn increase endogenous concentrations through cellular uptake.
